# The development of a public optometry system in Mozambique: a Cost Benefit Analysis

**DOI:** 10.1186/1472-6963-14-422

**Published:** 2014-09-23

**Authors:** Stephen Thompson, Kovin Naidoo, Geoff Harris, Luigi Bilotto, Jorge Ferrão, James Loughman

**Affiliations:** Dublin Institute of Technology, Dublin, Ireland/African Vision Research Institute, Durban, South Africa; African Vision Research Institute/Brien Holden Vision Institute, Durban, South Africa; Durban University of Technology, Durban, South Africa; Brien Holden Vision Institute, Sydney, Australia; Universidade Lúrio, Nampula, Mozambique

**Keywords:** Optometry, Cost benefit analysis, Health economics, Mozambique, Uncorrected refractive error, Eye health, Human resource development, Blindness, Visual impairment, Higher education

## Abstract

**Background:**

The economic burden of uncorrected refractive error (URE) is thought to be high in Mozambique, largely as a consequence of the lack of resources and systems to tackle this largely avoidable problem. The Mozambique Eyecare Project (MEP) has established the first optometry training and human resource deployment initiative to address the burden of URE in Lusophone Africa. The nature of the MEP programme provides the opportunity to determine, using Cost Benefit Analysis (CBA), whether investing in the establishment and delivery of a comprehensive system for optometry human resource development and public sector deployment is economically justifiable for Lusophone Africa.

**Methods:**

A CBA methodology was applied across the period 2009–2049. Costs associated with establishing and operating a school of optometry, and a programme to address uncorrected refractive error, were included. Benefits were calculated using a human capital approach to valuing sight. Disability weightings from the Global Burden of Disease study were applied. Costs were subtracted from benefits to provide the net societal benefit, which was discounted to provide the net present value using a 3% discount rate.

**Results:**

Using the most recently published disability weightings, the potential exists, through the correction of URE in 24.3 million potentially economically productive persons, to achieve a net present value societal benefit of up to $1.1 billion by 2049, at a Benefit-Cost ratio of 14:1. When CBA assumptions are varied as part of the sensitivity analysis, the results suggest the societal benefit could lie in the range of $649 million to $9.6 billion by 2049.

**Conclusion:**

This study demonstrates that a programme designed to address the burden of refractive error in Mozambique is economically justifiable in terms of the increased productivity that would result due to its implementation.

## Background

Refractive error occurs when the eye cannot clearly focus incident light on the retina, resulting in blurred vision. It refers principally to the conditions of myopia, hyperopia, and astigmatism, while presbyopia represents a related, and age dependent, inability to focus clearly on near objects. Uncorrected refractive error (URE) is the leading global cause of low vision, and causes almost half of all visual impairment (VI) [1]. It is recognised as a priority public health condition by a joint programme of the World Health Organization and the International Agency for the Prevention of Blindness under the global initiative, VISION 2020 [2]. Addressing URE is a priority, not just because of the burden of blindness and VI that it is responsible for, but also because of how easily and affordably it can be treated [3]. URE hampers education, limits employment opportunities, reduces productivity, and has been shown to impair quality of life [1, 3]. Simple and effective eye health interventions including an eye exam and provision of suitable spectacles can address the burden of URE [4].

In Mozambique, the burden of URE is thought to be high, having a severe impact on the livelihoods and wellbeing of disadvantaged communities [5]. A Rapid Assessment of Refractive Error (RARE) study in Mozambique found the prevalence of vision impairment was 3.5% (95% CI 49 2.7% - 4.2%), with 65.8% of those visually impaired being 35 years of age and older. URE prevalence was 2.6% (95% CI 2.1%-3.2%), and was the primary cause of vision impairment among 64.5% of cases. The spectacle coverage for URE was 0%. Presbyopia prevalence was higher, at 25.8% (95% CI 12.0% - 30.5%), with only 2.2% spectacle coverage [6]. By comparison, a RARE study completed in Eritrea found URE prevalence was 6.4% (95% CI 5.6%-7.2%) and spectacle coverage of 22.2%, while for presbyopia prevalence was 32.9% (95% CI 30.3%-35.7%) with spectacle coverage of 9.9% [7]. This comparison indicates how poorly developed and inaccessible refractive error services are in Mozambique compared to Eritrea.

The Mozambique Eyecare Project (MEP) is a partnership to facilitate the development, implementation and evaluation of the first and only optometry programme in Mozambique. This includes the establishment of a four-year BSc optometry course at Universidade Lúrio, a public university, in Nampula, northern Mozambique. Graduates are expected to work in the public health system once qualified. The recently founded programme presented a unique opportunity for Cost Benefit Analysis (CBA) to determine whether investing in optometry is economically justifiable.

Economic analysis is used to inform health planners and policy makers how limited resources should be allocated, indicating which interventions are good value for money. It can assist with justifying decisions on different resource allocation pathways [8]. CBA compares the resources spent on an intervention to the benefits gained or resources saved as a result of the intervention. It is useful for demonstrating savings associated with healthcare policy decisions [9], and can be used to gauge the desirability of an intervention in terms of its economic worth to society [10].

## Methods

Ethical approval was granted for the study under the Mozambique Eyecare Project (a joint initiative of Dublin Institute of Technology, University of Ulster, Universidade Lúrio and Brien Holden Vision Institute) by Dublin Institute of Technology, Republic of Ireland. Cost Benefit Analysis methodology was applied across the period 2009–2049. All costs were converted into United States Dollars ($). Costs were calculated using multiple resources including MEP financial reports, current market price information, and national human resources data. They included all costs associated with the establishment of an optometry degree programme, the establishment of vision centres within public hospitals, human resources costs, and overheads. The initial costs incurred during the implementation period were met by the MEP partners. Future costs may be met by the MEP partners, a range of funders, organisations interested to be involved in the partnership, and national government. The benefits associated with students enrolling in the last four years of the analysis period were omitted, although the costs associated with their education were included to reflect the on-going nature of the programme. Costs were subtracted from benefits to provide the net societal benefit, which was discounted to provide the net present value using a 3% discount rate to factor in the time value of money [11, 12]. Costs and benefits from years 2 through to 41 were discounted to the present value at the start of the program in 2009. Benefits were calculated using a human capital approach to valuing sight, measuring the potential economic productivity foregone by not addressing URE. Potential productivity gained by addressing URE was estimated using the value of $1,200 as Gross Domestic Product (GDP) adjusted for Purchasing Power Parity (PPP) per capita as a proxy indicator [13]. It was assumed that optometrists would work 242 days per annum, on average correcting the URE of 15 patients who are potentially economically productive per day (conservatively based on existing hospital clinic sessions staffed by ophthalmic technicians who examine 30–40 patients per day). A Labour Force Participation Rate (LFPR) of 82.75% and an Employment Rate (ER) of 79% were included to reflect the labour market and the fact that the emergence of productivity is not a certainty [13, 14].

The potential impact of visual impairment on productivity was calculated by applying disability weightings (DWs), used to quantify the severity of a disease or condition through a scale where 0 represents perfect health, 1 represents death, and every point in between reflects a level of disability associated with the specific disease or health condition, to PPP-adjusted GDP per capita. DWs for VI detailed in the 2010 Global Burden of Disease (GBD) study were incorporated into the analysis [15]. The original GBD study detailed a single DW for all categories of VI [11]. For the revised study, VI was divided into four categories [16], with each category assigned lower DWs than the original DW, as displayed in Table 1.

**Table 1:** VI category definition by visual acuity* in the better eye and associated new DWs

VI category	Definition	DW
Distance mild VI	<6/12 but ≥6/18	0.004
Distance moderate VI	<6/18 but ≥6/60	0.033
Distance severe VI	<6/60 but ≥3/60	0.191
Near VI	<6/12 but ≥3/60 for near, but ≥6/12 for distance	0.013

*Snellen visual acuity or the equivalent calculated from published LogMAR values.

Prevalence data of VI from previous studies informed distribution across the categories [17]. Due to limited data distinguishing between mild and moderate VI, these two categories combined into one and an average DW of 0.0185 assigned. To keep the analysis conservative, only the revised DWs for VI were used and the productivity loss associated with blindness (visual acuity < 3/60) was not included in the current study. Although the new DWs were used for the main body of this study, the original DW were included as part of the sensitivity analysis.

Spectacles were assumed to provide an effective solution to URE for up to four years once dispensed, based on studies in Africa, Asia, America and Europe [18, 19]. Further research is needed to establish whether this assumption is suitable specifically in the Mozambican context. To take into account prescription instability and spectacle frame/lens durability, effectiveness was assumed to be 100% in years one and two, 75% in year three and 50% in year four. After the fourth year the patient would return to suffering fully restricted productivity through URE and would need to return to the optometrist. The net benefit or loss was calculated by subtracting costs from benefits.

The population growth rate was assumed to stay constant at 2.3% per annum [20]. Seven students from the pilot group were assumed to enter the public system after graduation, with 15 expected to enter every subsequent year. The analysis period of 2009 to 2049 was defined by the time it would take, at such graduation rates, to reach a ratio of approximately one optometrist per 100,000 people, as shown in Table 2. This ratio represents half of the VISION 2020 target of one optometrist per 50,000 people [21]. Student numbers are already higher than those in this analysis but conservative graduation rates were selected to take into account death, career change, and decision not to work in the public sector.

**Table 2:** **Predicted population trends and number of optometrist graduates over time**

Year	2019	2029	2039	2049
Predicted population (million)*	28.7	36	45.2	56.8
Cumulative number of graduates	97	247	397	547
Optometrists per head of population	296,023	145,934	113,977	103,843

*Based on the population growth rate staying constant at 2.3% per annum [20].

## Results

### Costs

#### Expatriate lecturers costs

Expatriate lecturers were employed to teach until local human resources become available to take over. It was assumed 25 teaching years would be needed to reach this position. A staggered transition from expatriate to local faculty was assumed in order to ensure minimum impact to the students’ educational experience. The cost of $45,000 per annum per expatriate lecturer, based on actual incurred MEP costs, was included.

#### Mozambican lecturers costs

The first optometrists graduated in 2013, but those selected to teach will need additional pedagogic training before they are ready to be educators. The first Mozambican optometrist lecturers were scheduled to be employed in 2015, working alongside expatriate lecturers during a transition period. By 2017 all lecturers were assumed to be Mozambican, at a cost of $18,514 per annum per lecturer.

#### Management costs

MEP Management costs included advocacy, project and financial management, procurement, research and human resource development. Costs were $1.7 million over the first six years, to reflect the assigned Programme for Strategic Cooperation funding from Irish Aid and the project partners. A rate of $100,000 per annum was applied thereafter, to reflect on-going support from the partners, which will depend on securing new funding once the original funding phase has ended.

#### School equipment costs

In the first four years $330,000 was spent on the purchase and maintenance of equipment, with a rolling cost of $50,000 per annum applied thereafter.

#### Educational material costs

During the first four years, the actual cost of developing and translating educational materials was $126,000, with $5,000 per annum every year thereafter.

#### Book/academic literature costs

The cost of $3,000 per annum was assigned for books and academic literature.

#### Faculty operating costs

To reflect the general cost of running the university, $800 per student per annum was included (university estimate).

#### Vision centre equipment costs

Once graduated, it was assumed that optometrists would work in Vision Centres, installed in existing public health facilities. A Vision Centre, designed to support two optometrists was calculated to cost $45,250. Costs included equipment, refurbishment/renovations, transport including shipping and customs, management time, support costs, monitoring and evaluation and project management costs.

#### Vision centre human resource costs (Optometrists)

The public sector salary of an optometrist with less than three years of experience was confirmed as $9,528 per annum, increasing to $9,912 per annum after three years of service.

#### Vision centre human resources costs (Technicians)

Each location was also assumed to require a technician to support the clinics, maintain equipment, and to assist with the manufacturing of spectacles bespoke to each patient. The salary of $3,503 per employee per annum was included.

#### Vision centre human resources costs (Administrators)

Each location was also assumed to require one administrator, who would receive the salary of $3,503 per annum.

#### Vision centre overheads costs

For use of electricity, water, and basic upkeep of the building, $672 per optometrist per annum was included. Significant building repair have not been included in this analysis.

Between 2009 and 2049, after applying a 3% discount rate the net present value of the cost of training and employing optometrists was $83.9 million, as shown in Table 3. The salaries of the optometrists represented by far the highest proportion of costs, accounting for 54.1% of the total.

**Table 3:** **Net present value of costs 2009 – 2049 after applying a 3% discount rate**

Cost	$	%
Expatriate teaching faculty	1,013,912	1.2
Local teaching faculty	1,669,727	2.0
Programme management costs	3,419,385	4.1
School equipment cost	1,329,116	1.6
Educational material costs	221,578	0.3
Book costs	72,344	0.1
General faculty operating costs	4,753,419	5.7
Vision centre equipment costs	6,737,416	8.0
Vision centre human resources costs (optometrists)	45,404,417	54.1
Vision centre human resources costs (technicians)	8,099,332	9.7
Vision centre human resources costs (administrators)	8,099,332	9.7
Overheads	3,100,461	3.7
**Total**	83,920,439	100.0

### Benefits

Economic benefits are not realised until the fifth year (2013) when the first optometrists graduate and enter the public health system. By 2049, after applying a 3% discount rate, a net present value gross societal benefit $1.2 billion using the new DWs. This represents the value of correcting the URE of 24.3 million patients who are potentially economically productive.

### Cost benefit analysis

**Figure 1:**
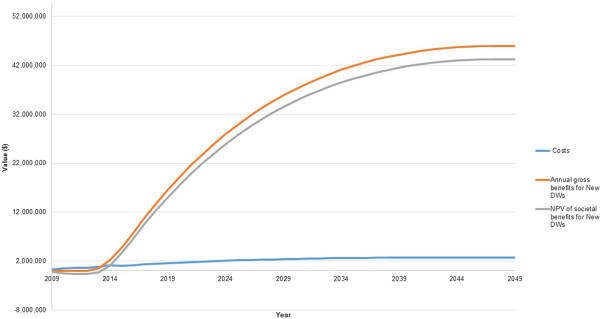
**Costs and benefits of the optometry programme with 3% discounting.**

Using the new DWs, the present value of the annual net societal benefit is negative until 2014. From 2014 there is a positive annual net societal benefit. This continues until the end of the analysis. By 2049, a net present value of $1.1 billion in societal benefits is realised. The results are illustrated in Figure 1.

The Benefit-Cost Ratio (BCR) can be used to evaluate the economic merit of a programme. A ratio where the benefits are greater than 1 suggests the programme is economically justifiable. Using the new DWs after discounting, the BCR is 14:1. The cumulative net societal benefits are positive by 2049. Initial costs associated with implementing the programme are eclipsed by the much higher benefits realised in later years.

### Sensitivity analysis

Methodological sensitivity to the discount rate was tested, and the results indicated that the outcome of the study did not change until a 72% discount rate was applied, at which point the net present value of societal benefits for new DWs became negative. Without any discounting, a total of $2.5 billion in societal benefits are recorded by 2049.

Further investigation into the sensitivity of the methodology was implemented by comparing four scenarios based on different assumptions. Scenario 1 was the most conservative in comparison to the other three. It assumed that spectacles would only be effective for two years instead of four years and the salary of optometrists was doubled. It employed the revised DWs. Scenario 2 employed the same assumptions as scenario 1, but maintained the salaries at the original level detailed in the main body of the study. Scenario 3 represented the assumptions used in the main analysis and were included to allow comparison. Scenario 4 was the least conservative. It employed the same assumptions as scenario 3, but used the original DW as opposed to the new DWs.

**Figure 2:**
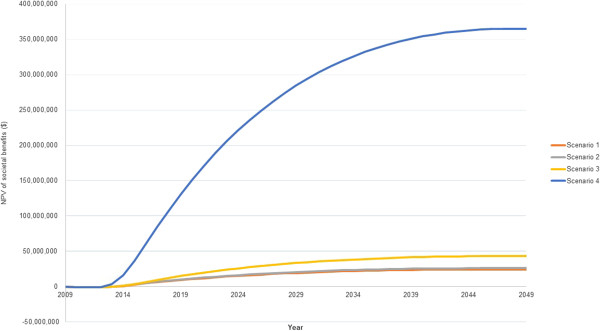
**The results of the sensitivity analysis, showing the NPV of societal benefits ($) at 3% discount rate.**

Scenario 1 found that by 2049, $649 million in societal benefits would be realised. Scenario 2 found that by 2049, $695 million in societal benefits would be realised. As described in the main analysis, scenario 3 resulted in a net societal benefit of $1.1 billion by 1949. For the first 3 scenarios the annual net societal benefit is negative until 2013 and positive from 2014 until the end of the time period analysed. Scenario 4 found that the annual net societal benefit is negative until 2012. From 2013 until the end of the time period analysed, it is positive. By 2049, a net present value of $9.6 billion of societal benefits will have been realised. Figure 2 illustrates the results of the sensitivity analysis, showing the NPV of societal benefits at 3% discount rate for the four scenarios analysed.

The sensitivity analysis found that regardless of parameter changes to assumptions made about the costs, benefits or disability weighting, a positive net societal benefit is realised very soon after the optometrists graduate and commence work. Even when assumptions were extremely conservative, $649 million in societal benefits could be realised by 2049. When less conservative assumptions were used, the model found that $9.6 billion in societal benefits could be realised by 2049.

## Discussion

During the early years of the programme, funds are spent to establish the optometry school but no benefits are realised until the first optometrists start work. This period of high costs and no return is to be expected as a normal implementation phase for any human resources development programme. In the years following the implementation phase, the annual BCRs indicate a positive return on investment. With competition for limited resources and funds coming from both outside and within health systems, optometry is shown to be worthy of investment due to the potential for societal benefit. The results complement those of existing literature, which found interventions that address URE to be economically justifiable [1, 4, 17, 22].

The new DWs for blindness and VI have been the subject of much debate. The Vision Loss Expert Group (VLEG), comprising of leading ophthalmologists and optometrists, has expressed concern at how low the new DWs are compared to the previous weighting, particularly noting variations in the formulation of the lay survey questions pertaining to different health conditions. Vision and hearing loss are notable outliers in the 2010 GBD study. When compared to the assigned DWs for moderate skin disfigurement with itch or pain (0.187), mild alcoholism (0.259), moderate rheumatoid arthritis (0.292), or even a pain in the neck (0.221), the VLEG authors note that the new DWs for blindness and VI simply do not pass the “common sense test”. VLEG calls for the new DWs to be investigated further prior to their widespread adoption [23]. The GBD authors have defended the new DWs, by stating that the definitions of blindness and VI were informed through consultation with VLEG and that the weights were informed by a series of measurement surveys, in which 30,000 people participated. They also issued a warning not to use previous weights as if they were a reference standard [24]. The results of this study exemplify the impact that revising DWs can have on the assessment of an intervention. In the case of the burden of URE as addressed herein, the range of societal benefits that are projected vary nine fold, from $1.1 billion for the revised DWs to $9.6 billion for the original DW. Many CBA methodologies are available, and different theories exist in relation to which costs and benefits to include. In the current study no building costs were included as the Universidade Lúrio campus was already established. Also the costs of lenses and frames were excluded, as the spectacles would be sold at least for a nominal fee to cover costs. To keep the analysis conservative, net profit from sale of spectacles was not included. This revenue has potential to contribute not only to the economic stability and sustainability of the refraction service, but of a complete eye health department [17, 25, 26].

The current study does not address people’s willingness to pay, ability to pay or any barriers to access for a refractive service. Quality of delivery, as well as supply and distribution processes will determine how successful the programme is [4, 27]. Further research on this is needed. Acceptance is also a factor. The compliance ratio has a direct relationship with the benefits realised. The study assumes 100% spectacle wear compliance for patients with refractive error, which may not be the case. If half of the patients rejected the spectacles or took them but never wore them, then the benefits would also be reduced by half. Also, addressing URE does not guarantee the emergence of economic productivity. The conditions and environment will need to be conducive to economic opportunity or entrepreneurial opportunity for productivity to flourish after URE is addressed.

As in any CBA study, judgements were made concerning the inclusion of cost and benefit items and assumptions were made concerning their size and/or incidence. For the base case scenario, these judgements were always conservative and so reduced the estimated societal benefit. The sensitivity analysis tested the robustness of the methodology against changing assumptions.

Inflation costs were deliberately excluded. In periods of inflation, market prices and costs do not measure true values of benefits and costs. The values of benefits and costs appear to increase although their true value does not. Real values are values with the purchasing power of money held constant relative to a specific point in time [10].

This study builds on existing literature, in which economic analyses have been employed to estimate the value derived from addressing blindness and VI. One study found that addressing URE has the potential for the greatest impact on the global economy compared to all other preventable vision disorders [28]. Another found investing in eye health in The Gambia to be economically justifiable and a third found that globally, in excess of $100 billion in lost productivity could be avoided if all the targets set by VISION 2020 are achieved for the period from 2003 to 2020 [11, 12]. Focusing exclusively on the impact of VI caused by URE on economic productivity, it was estimated that the global loss associated with this burden was $268.8 billion after adjustment for LFPR and ER. While direct comparison between studies is challenging due to differences in scale and scope, both the global study and the current national level study for Mozambique provide sound economic reasoning to invest in interventions that address URE and reduce the burden of VI [28].

The study assumes that the graduation rate remains constant after the pilot year. In reality, the number of students and optometrists will increase as the course becomes more established and the cadre develops. The enrolment rate in the study was deliberately conservatively low. If enrolment increased, so would some costs, but not all. For example, educational material costs would stay the same, but school equipment costs would need to increase in proportion to the number of students. If the CBA model is updated to assume double the number of students graduate each year, to represent the VISION 2020 target of one optometrist per 50,000 people, subject to a 3% discount rate, a net present value societal benefit of $2.3 billion when using the new DWs could be realised by 2049. This represents the value of correcting the URE of 48.6 million patients who are potentially economically productive by 2049. The annual BCR remains virtually static as an increase in students and graduates increases both the costs and benefits.

The focus of the current study is URE but optometrists can also reduce the burden of blindness and VI caused by other diseases and conditions - the benefits of which are not reported here. Blindness and VI are further associated with additional indirect health effects and costs. An effective programme to address avoidable vision loss will generate cost savings, as, for example, the rates of falls, fractures, motor vehicle accidents and conditions such as depression attributable to low vision are reduced. Premature death due to blindness and VI would also result in a future stream of productivity losses due to lost potential earnings. Such indirect cost savings and productivity gains are difficult to accurately quantify, and, therefore, not included in the model, but would serve to increase the net societal benefit.

Although URE is also a cause of blindness (visual acuity < 3/60) which is afforded higher DWs relative to VI (Original DWs, 0.6; revised DWs 0.195), the prevalence data on blindness associated with URE is inconsistent, ranging from 1.1% to 7.9% in some studies, but with more than half of all studies across sub-Saharan Africa reporting zero blindness as a consequence of URE [29]. Due to the likely low overall blindness prevalence as a result of URE, the current study includes visual impairment only, which reinforces the conservative nature of the societal benefit estimate.

Also, the burden of URE is, in reality, not limited to just the individual who is visually impaired. Families, societies and communities may also suffer the burden as they may be required to give up their time to perform certain tasks to assist or care for the person with VI. One study assumed that for each blind individual, a 10% loss of productivity would be experienced by a relative or someone in the community [11]. If the wider societal burden were to be included, net societal benefits of addressing the URE would increase. The current study focuses on people of productive age. It does not consider that if the URE of a child is addressed, this would be expected to maximise their future economic productivity through improved education and consequentially enhanced employment opportunities.

## Conclusion

The development of optometry has been shown to have the potential to achieve a net present value societal benefit of $1.1 billion by 2049 in Mozambique using conservative DWs and after applying a 3% discount rate. Investment in optometry is shown to be attractive and justifiable in economic terms. When CBA assumptions are varied as part of the sensitivity analysis, the results suggest the societal benefit could lie in the range of $649 million to $9.6 billion by 2049, depending on how conservative the assumptions are. While costs can be justified, as they are far outweighed by the benefits optometrists bring, they still present a large cash outflow for the public health sector of a low income country. Careful planning is needed to ensure the budget is available to employ the optometrists once trained within the public sector. If the budgetary resources are not available to pay the optometrists wages, they will either seek employment in the private sector or possibly look to emigrate. Either of these scenarios would result in the burden of URE in Mozambique remaining high.

To conclude, the results reinforce observations from other national and international eye health studies that suggest that investment in eye health, and particularly programmes that address URE, provide good value for money and can be justified economically.

## Abbreviations

BCR: Benefit-cost ratio
CBA: Cost benefit analysis
DW: Disability weighting
ER: Employment rate
GBD: Global Burden of Disease
GDP: Gross Domestic Product
LFPR: Labour Force Participation Rate
MEP: Mozambique Eyecare Project
NPV: Net present value
PPP: Purchasing power parity
URE: Uncorrected refractive error
VI: visual impairment
VLEG: The Vision Loss Expert Group.
